# Surfactant Protein-C Regulates Alveolar Type 2 Epithelial Cell Lineages via the CD74 Receptor

**DOI:** 10.70322/jrbtm.2024.10017

**Published:** 2024-10-11

**Authors:** Krishan G. Jain, Yang Liu, Runzhen Zhao, Preeti J. Muire, Nan-Miles Xi, Hong-Long Ji

**Affiliations:** 1 Department of Surgery, Stritch School of Medicine, Loyola University Chicago, Maywood, IL 60153, USA; 2 Burn and Shock Trauma Research Institute, Stritch School of Medicine, Loyola University Chicago, Maywood, IL 60153, USA; 3 Department of Orthopedics and Rehabilitation, Stritch School of Medicine, Loyola University Chicago, Maywood, IL 60153, USA; 4 Infectious Diseases and Immunology Research Institute, Stritch School of Medicine, Loyola University Chicago, Maywood, IL 60153, USA; 5 Department of Biostatistics and Data Science, University of Texas Medical Branch, Galveston, TX 77550, USA

**Keywords:** Alveolar type 2 cell, Surfactant protein-C, ARDS, Differentiation, Proliferation, Re-alveolarization

## Abstract

**Background:**

Deficiency of surfactant protein-C (SPC) increases susceptibility to lung infections and injury, and suppressed expression of SPC has been associated with the severity of acute respiratory distress syndrome (ARDS). Alveolar type 2 epithelial cells (AT2) are critical for maintenance and repair of the lung. However, the role of the SPC in the regulation of AT2 cell lineage and the underlying mechanisms are not completely understood.

**Methods:**

This study aimed to investigate the mechanisms by which SPC regulates AT2 lineages. *Sftpc−/−* mice were used to model the SPC deficiency in ARDS patients. We utilized three-dimensional (3D) organoids to compare AT2 lineage characteristics between wild type (WT) and *Sftpc−/−* mice by analyzing AT2 proliferation, alveolar type 1 cells (AT1) differentiation and CD74 expression, using colony-formation assay, immunofluorescence, flow cytometry, and immunoblots.

**Results:**

The results showed that *Sftpc*−/− mice demonstrated a reduced AT2 cell population. Influenza A virus subtype H1N1 (H1N1) infected *Sftpc−/−* mice demonstrated reduced AT2 proliferation and AT1 differentiation. Western blot indicated elevated levels of CD74 protein in AT2 cells of *Sftpc−/−* mice. Colony-forming efficiency was significantly attenuated in AT2 cells isolated from *Sftpc−/−* mice compared to the WT controls. Podoplanin (PDPN, a marker of AT1 cells) expression and transient cell count significantly increased in *Sftpc−/−* organoids. Moreover, siRNA-mediated gene silencing of CD74 in AT2 cells significantly increased AT2 proliferation and AT1 differentiation in *Sftpc−/−* organoids.

**Conclusions:**

This study suggests that SPC regulates AT2 lineage in vitro and in vivo. The SPC might influence AT2 lineage during the lung epithelium repair by activating signaling mechanism involving CD74 receptor.

## 1. Introduction

Acute respiratory distress syndrome (ARDS) is a condition characterized by increased permeability of the alveolar epithelium. This hyperpermeability is primarily caused by the disrupted epithelial layer of the blood gas barrier. These disruptions can be triggered by viral infections such as severe acute respiratory syndrome coronavirus 2 (SARS-CoV-2) and influenza, as well as by inflammatory and coagulation processes [1–3]. The damage to the alveolar epithelium activates AT2 cells, which undergo proliferation and differentiation into mature AT1 cells to repair the injured alveolar epithelium. This process helps regenerate the epithelial barrier and restore normal lung function [3,4].

The AT2 cells express a protein called surfactant protein-C (SPC), which is highly hydrophobic [5]. Its primary function is to form a protective surfactant layer at the air-liquid interface of the alveoli. This surfactant film reduces alveolar surface tension, enabling alveoli to remain inflated and facilitating efficient gas exchange [6]. It is well established that AT2 cell death correlates with reduced SPC levels. This decreased SPC expression leads to increased alveolar surface tension, potentially hindering gas exchange in the lungs [7]. Reduced levels of SPC are associated with various lung diseases, including familial interstitial lung disease (ILD), neonatal respiratory distress syndrome (NRDS), idiopathic pulmonary fibrosis (IPF), and ARDS in some patients [8–12]. Coronavirus disease 2019 (COVID-19) may also affect SPC levels and lung function [13,14]. Reduced SPC might impair lung repair by impacting AT2 cell proliferation and AT2 to AT1 differentiation. New research has identified a peculiar stage of AT2 cells in damaged lungs. These cells show signs of halting division (cell-cycle arrest), a loss of their typical AT2 characteristics, and a slight increase in markers associated with AT1 cells. This evidence suggests that a buildup of these “in-between” AT2 cells, unable to fully transform into AT1 cells, might be a key reason why lung repair fails in IPF patients [15]. In one case, exogenous surfactant treatment improved oxygen levels in a COVID-19 patient with ARDS, suggesting a potential therapeutic approach [16]. However, the role that the *Sftpc* gene plays in regulating re-alveolarization after lung injury remains unclear.

CD74 is a type II transmembrane protein that is a chaperone for major histocompatibility complex class II (MHCII). CD74 is expressed in AT2 cells in humans and mice [17–19]. CD74 has been approved as a biomarker for ICU-acquired infections [20], bronchopulmonary dysplasia [21], and lung injury [22]. Recent research on lung injury has shown that CD74 expression increases in patients with acute lung injury (ALI) and COVID-19 [23,24]. CD74 contributes to host defense against SARS-CoV-2 and other virus entry [25,26]. H1N1 and H3N2 influenza infections resulted in reduced expression of both SPC and CD74 in AT2 cells [17]. However, CD74 was upregulated in human AT2-originated adenocarcinoma [18]. Whether CD74 is involved in the AT2 lineage regulation is completely unclear. We hypothesize that the CD74 receptor triggered by SPC plays a crucial role in directing AT2 cell lineage. Disruption of this signaling could lead to impaired functionality of AT2 cells, potentially increasing susceptibility to lung infections and injuries. This study utilized a 3D organoid model to investigate the mechanism for the regulation of primary AT2 cell lineage by SPC and CD74 receptors.

## 2. Materials and Methods

Aim: This study aimed to investigate the mechanisms by which SPC regulates AT2 lineages.

Antibodies and kit details are available in the online supplementary material (Table S1).

### 2.1. Animals

The wild type (WT) 129S6 mice were purchased from Taconic Biosciences. The *Sftpc−/−* mice, bred on a 129S6 background, were acquired from Dr. Timothy E. Weaver and Dr. Jeffrey A. Whitsett in the Department of Pediatrics, Cincinnati Children’s Hospital Medical Center. All mice were housed in a pathogen-free facility with a 12 h light/dark cycle, and they had ad libitum access to food and water. Paired WT and *Sftpc−/−* mice, age 2–4 months, both male and female, were used for experiments. These procedures were conducted following approval from the Institutional Animal Care and Use Committee of the University of Texas at Tyler Health Science Center.

### 2.2. Fluorescence-Activated Cell Sorting (FACS) Mediated AT2 Cell Isolation

AT2 cells from mice were isolated utilizing a previously published method with some modifications [27,28]. After inducing anesthesia with ketamine/xylazine (100/8.5 mg/kg), the mouse’s abdominal aorta was cut off to exsanguinate the animal. Subsequently, the lung underwent perfusion with Dulbecco’s Phosphate Buffered Saline (DPBS) through the right ventricle to eliminate blood, followed by infusion with a 50 U/mL dispase solution through the trachea. A low melting point agarose was injected into the lung to prevent leakage of the dispase solution. Before enzymatic dissociation, we carefully removed the trachea and large airways, using only lung lobes for AT2 isolation. This step excludes airway epithelial cells from the preparation. Only AT2 and AT1 epithelial cells are present in the lung lobes. The lung lobes, filled with dispase, were then incubated in a 50 U/mL dispase solution for 45 min at 25 °C. Cells were released from the lung by delicately teasing it in DMEM/F12 containing 0.01% DNase I. Furthermore, the AT1 cells in vivo are large, flattened and very fragile, often fragmenting during the enzymatic and mechanical processing of lung lobes. We removed blood cells using magnetic sorting before FACS sorting for 7AAD^−^/EPCAM^+^ cell AT2 cells. After passing through 100 μm, 40 μm, and 10 μm cell strainers, cells were labeled with biotin-conjugated CD16/32 (BD Biosciences, San Jose, CA, USA #553143), CD45 (BD Biosciences #553078), and TER119 (BD Biosciences #553672) antibodies. Streptavidin-coated magnetic beads (Invitrogen, Carlsbad, CA, USA; #65601) were employed to negatively select AT2 cells. To enhance the purity and viability of AT2 cells, the cells were labeled with AF488-conjugated epithelial cell adhesion molecule (CD326/EPCAM) antibody (Biolegend, San Diego, CA, USA, #118210) and 7-AAD viability dye. FACS was employed to sort EPCAM^+^ and 7-AAD^−^AT2 cells, ensuring a collection with over 98% viability and purity. FlowJo v10.9 software was used to analyze FACS data. We first gated all the 7-AAD^−^ negative live cells. Then, from the live cells, we gated EPCAM^+^ AT2 cells.

### 2.3. Feeder-Free Culture of Alveolar Organoids

EPCAM^+^ AT2 cells, sorted through FACS, were cultured in Matrigel to generate organoids, as previously outlined protocol with some modifications [28,29]. In brief, AT2 cells were combined with growth factor-reduced Matrigel (Corning, Corning, NY, USA, #354230) or Cultrex RGF Basement Membrane Extract, Type 2 (R&D Systems #3533-010-02P) and diluted with organoid growth medium (AMM) at a 1:1 ratio to create suspensions containing 30–100 cells/μL [28]. 60 μL of the cell suspension was added to the apical chamber of transwell inserts (Corning #3470), or 150 μL of suspension was added onto a well of a 6-well plate to form 10 droplets. After a 30-min incubation at 37 °C for the matrix to solidify, 500 μL or 1500 μL of AMM with 10 ng/mL IL-1β and 10 μM Y-27632 was introduced into the bottom well of the transwell or the well of the 6-well plate. After 4 days of incubation, the medium was switched to AMM without IL-1β and Y-27632 and changed every 3 days. On day 10, the organoids were subjected to differentiation, collected for sampling, or passaged for expansion. For AT1 differentiation, AMM was substituted with organoid differentiation medium (ADM) [28], and the organoids were maintained for 7 days. Colonies were observed after a 10-day proliferation period and a 17-day differentiation phase, and brightfield images were captured using an Evos XL core microscope (Life Technologies, Carlsbad, CA, USA) with a 2× microscope objective. Colony number and colony-forming efficiency of organoids were analyzed using ImageJ software [30].

### 2.4. Western Blotting

Western blotting was performed using standard methods. Lung tissue, organoids or AT2 cells were lysed in ice-cold RIPA buffer containing protease and phosphatase inhibitors. Protein concentrations were determined using a BCA protein assay (Pierce, Waltham, MA, USA, #23225). The protein samples were added to 4× loading buffer and boiled at 95 °C for 10 min. 10–25 μg of the proteins were then separated using an SDS-PAGE gel. After electrophoretic separation, proteins were transferred to PVDF membranes. The membranes were blocked with 5% non-fat milk and incubated with primary antibodies at 4 °C overnight. The primary antibodies were used as follows: anti-SPB rabbit polyclonal antibody (ThermoFisher, Waltham, MA, USA, #PA5-42000, 1:1000), anti-PDPN Syrian hamster antibody (Invitrogen MA516113, 1:1000), anti-proSPC rabbit antibody (Millipore, Burlington, MA, USA, #AB3786, 1:500), anti-CD74 mouse antibody (Santa Cruz Biotech, Dallas, TX, USA, #SC-6262) and anti-β-actin mouse monoclonal antibody (Santa Cruz Biotech #sc-47778, 1:1000) were diluted in 5% non-fat milk. HRP-conjugated goat anti-mouse IgG (Jackson ImmunoResearch, West Grove, PA, USA, #115-035-147, 1:10,000), mouse anti-rabbit IgG (Jackson ImmunoResearch #211-032-171, 1:10,000), and goat anti-Syrian hamster IgG (Jackson ImmunoResearch #107-035-142, 1:10,000) were used as the secondary antibodies. Blots were visualized with chemiluminescence (Millipore #WBKLS0500) using a Bio-Rad Chemidoc imaging system. Images were analyzed using ImageJ software.

### 2.5. Immunofluorescent Staining

Organoids were fixed in 4% paraformaldehyde for 1 h at room temperature and washed twice with PBS containing 0.1% BSA. For permeabilization and blocking, organoids were treated with PBS containing 0.3% Triton X-100, 3% BSA, and 5% goat serum for 1 h. Primary antibodies, including anti-SPB rabbit polyclonal antibody (ThermoFisher #PA5-42000, 1:200) and anti-PDPN Syrian hamster mouse antibody (Invitrogen MA516113, 1:500), were diluted in PBS containing 3% BSA and 5% goat serum, and then added to the slides for overnight incubation at 4 °C. Secondary antibodies, AF 488 goat anti-rabbit IgG (H + L) (Jackson ImmunoResearch #111-545-045, 1:500) and AF647 Goat anti-Syrian Hamster IgG (H + L) (Invitrogen #A-21451, 1:500), along with DAPI (1:1000), were applied for 1 h incubation at room temperature. The slides were mounted with mounting medium (Electron Microscopy Sciences, Hatfield, PA, USA, Cat#17985-10) and sealed with nail polish. Images were captured using a Zeiss LSM 510 confocal microscope or Zeiss Axiovert 200M microscope. Subsequently, all images were processed and analyzed using ImageJ software.

### 2.6. Mice Infection with H1N1 Virus

Age and sex-matched WT 129S6 and *Sftpc−/−* mice (*n* = 5) were anesthetized with ketamine (100 mg/kg)/xylazine (8.5 mg/kg). Each mouse was intranasally inoculated with 50 μL of PBS or 7320 pfu of H1N1 (A/PR/8/34, Charles River) as previously described [31]. Mice were closely monitored until they were fully recovered and then returned to their cage. Their weight and health were monitored daily. Mice with over 20% weight loss were humanely euthanized according to the approved protocol by IACUC. Lungs were perfused with PBS and processed for flow cytometry and Western blot analysis. All H1N1 work was performed in a Class II biosafety cabinet under BSL2 conditions.

### 2.7. Flow Cytometry

The single-cell suspension was prepared from lungs and organoids as previously described for flow cytometry analysis of AT2 (SPB^+^), AT1(RAGE^+^), and transitional (SPB^+^ and RAGE^+^) cells. Organoids were dissociated enzymatically with TrypLETM Select (Gibco, New York, NY, USA, #12563-029) and passed through a 40 μm cell strainer to prepare a single cell suspension. Cells were fixed and permeabilized using BD Cytofix/Cytoperm^™^ Fixation/Permeabilization Kit (BD#554714). Cells were labeled with anti-SPB rabbit polyclonal antibody (ThermoFisher #PA5-42000, 1 ug/million cells) and AF647-conjugated anti-RAGE antibody (R&D systems FAB11795R, 1 ug/million cells). AF488-conjugated goat anti-rabbit IgG (H + L) (Jackson ImmunoResearch #111-545-045) secondary antibody was used to detect the SPB signal. Cells were analyzed using a BD Fortessa flow cytometer. Unstained and single-color stained samples were used as controls for gating. Data was analyzed using FlowJo v10.9 software.

### 2.8. EdU Labeling of Proliferating AT2 Cells

To identify actively proliferating AT2 cells in organoids, the Click-iT 5-ethynyl-2-deoxyuridine (EdU) assay kit (ThermoFisher #C10499) was employed. Organoids were incubated in a medium containing 10 mM EdU for 3 h and then fixed with 4% paraformaldehyde for subsequent staining with the Click-iT EdU AF488, following the manufacturer’s instructions. Confocal microscope images were captured and analyzed to determine the percentage of EdU-positive cells. The z-sections of an entire organoid were stacked to count the total number of cells (DAPI-stained nucleus) and EdU^+^ cells (AF488 signal) using a cell counter plug-in for ImageJ. The percentage of EdU^+^ cells was calculated and compared between WT and *Sftpc−/−* samples.

### 2.9. siRNA-Mediated Knockdown of CD74

Proliferative organoids were dissociated to prepare a single-cell suspension of AT2 cells. AT2 cells (1 × 10^6^ per well) were seeded in 6 well plates pre-coated with Matrigel (1:1 with AMM) and incubated overnight for attachment. The following day, the media was removed, and 80 pmol CD74 siRNA (Santa Cruz Biotechnology, Dallas, TX, USA, #SC-35024) or scramble (SCR) control (Santa Cruz Biotechnology #SC-37007) was added to the cells for 6 h and 24 h. After 6 h or 24 h, 2 mL of AMM was added to the wells and incubated overnight. Next day, cells were collected for WB to confirm CD74 knockdown and for growing organoids to analyze the effect of CD74 knockdown on organoid colony formation and AT1 differentiation. We repeated siRNA and scramble treatments in the culture after 10 days to inhibit CD74 expression throughout the culture for 17 days.

### 2.10. Statistics Analysis

All results are reported as mean ±SD or mean ±SEM. Comparisons between WT and *Sftpc−/−* mice were made using two-tailed student *t*-tests and between WT scrambled, *Sftpc−/−* scrambled, and *Sftpc−/−* siRNA groups were conducted using one-way ANOVA, with *p* < 0.05 considered significant. Statistical tests were conducted using GraphPad Prism10 software.

## 3. Results

### 3.1. Sftpc Gene Knockout Reduced AT2 Population

**Figure 1.**
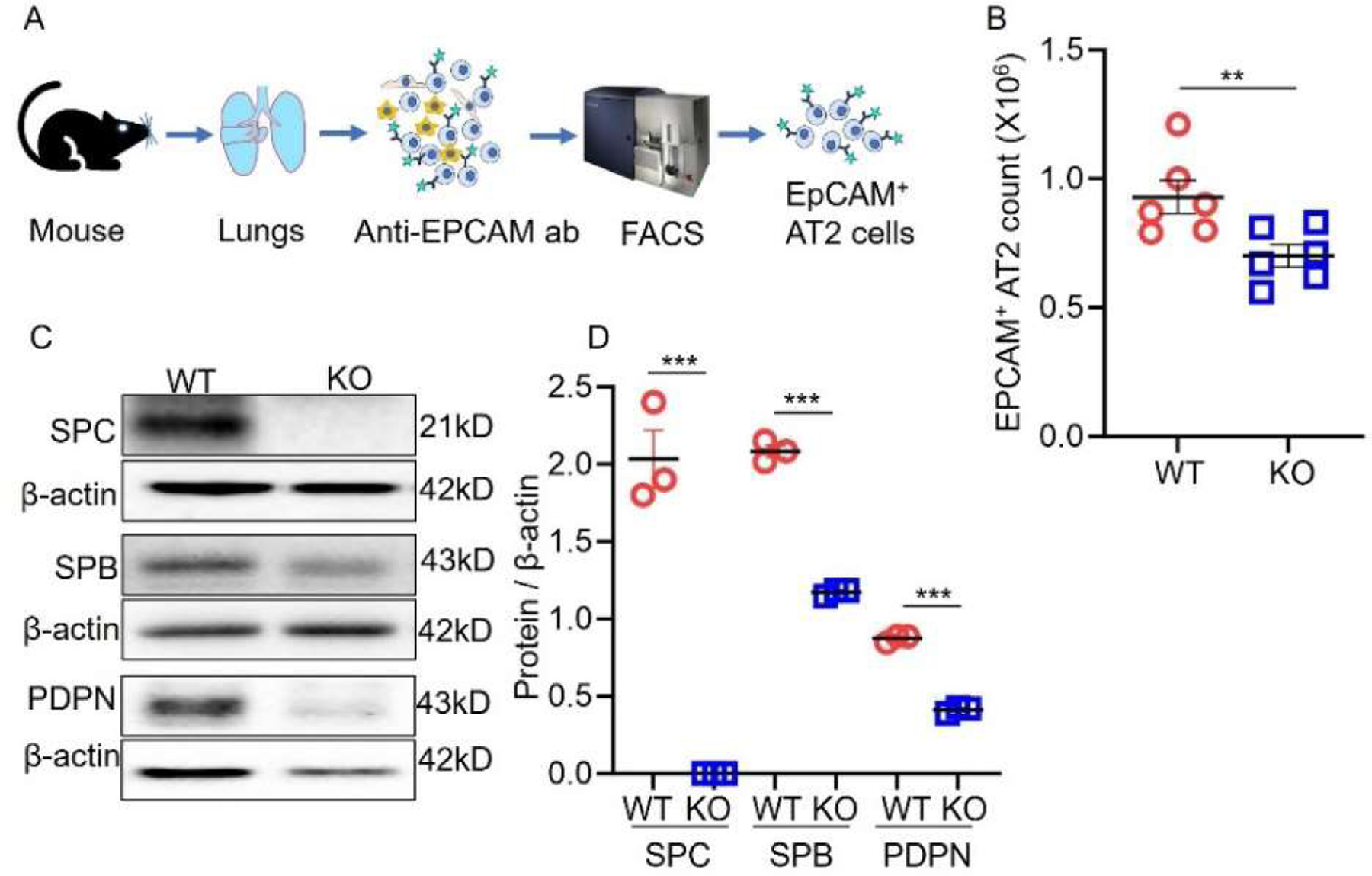
SPC deficiency reduced AT2 population in vivo. (**A**) Procedure of EPCAM^+^ AT2 cell isolation using FACS. ab, antibody. (**B**) Scatter dot plot for the total EpCAM^+^ AT2 population. 2-tailed Student *t*-test, ** *p* < 0.01. *n* = 6. (**C**) Representative Western blots from whole lung lysate. Blots were cropped to improve the clarity. The expression of AT2 markers (SPC and SPB) and AT1 marker (PDPN) in WT and *Sftpc−/−* lungs (KO). (**D**) Protein expression levels cell markers. Data are presented as mean ± SEM. 2-tailed Student *t*-test, *** *p* < 0.001. *n* = 3.

To investigate the effects of SPC deficiency on AT2 cells, EPCAM^+^ AT2 cells were isolated from the lungs of WT and *Sftpc−/−* mice (*n* = 6 mice/group). Our findings revealed a significant decrease in the total number of EPCAM^+^ AT2 cells obtained from *Sftpc−/−* mice compared to the WT control group (Figure 1B). This suggests that the absence of the *Sftpc* gene negatively impacts the overall population of EPCAM^+^AT2 cells. Western blot analysis was performed on homogenized lung tissue from these mice (Figure 1C) to further characterize protein expression. As expected, the SPC protein was completely absent in *Sftpc−/−* mice compared to WT controls (Figure 1D). Additionally, we observed a significant decrease in SPB and PDPN protein levels in the lungs of *Sftpc−/−* mice (Figure 1D). Full-sized blots are available as Figure S1 in Supplementary Materials.

### 3.2. H1N1 Infection Increased Transitional Cells in Sftpc−/− Mice

**Figure 2.**
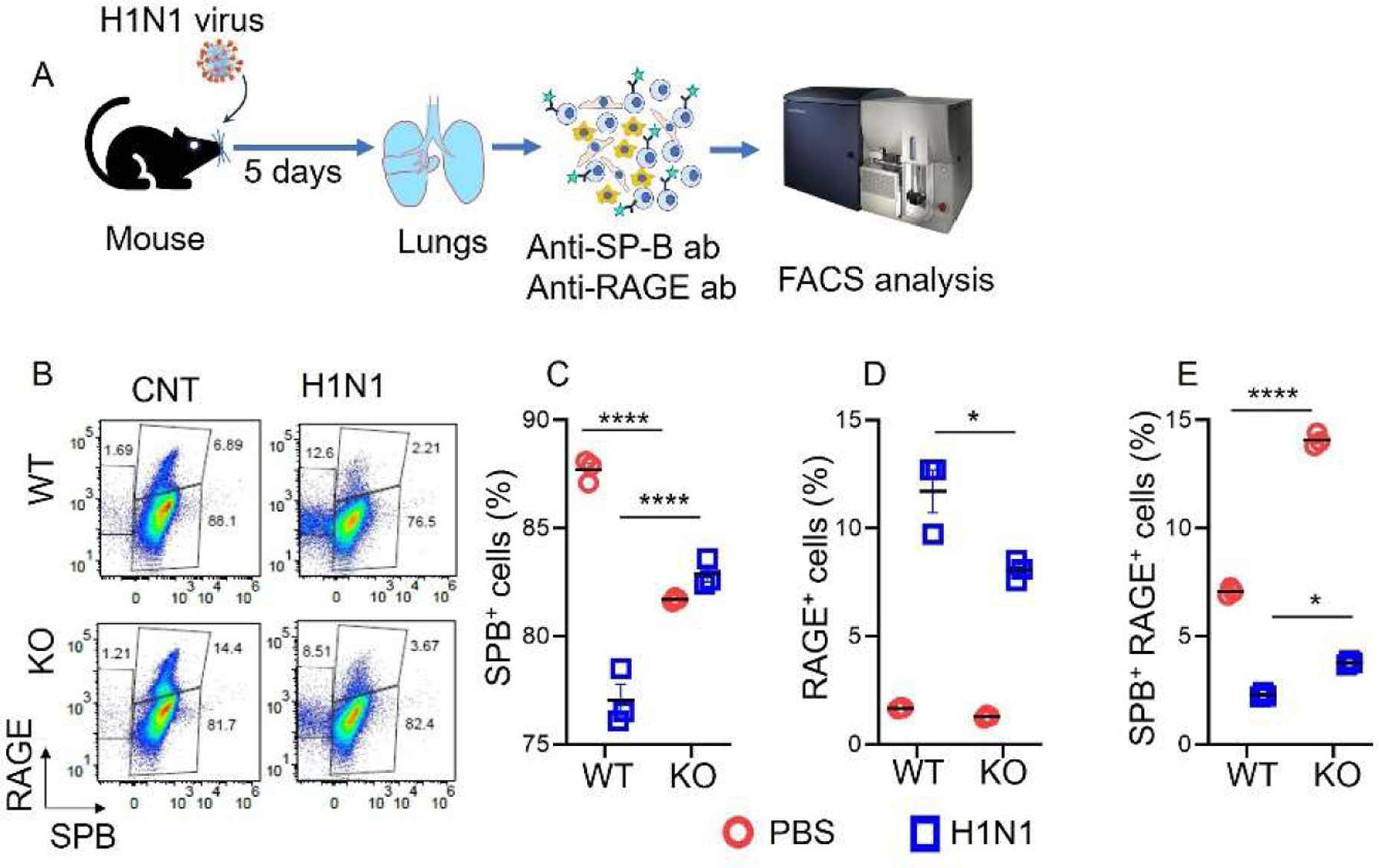
SPC deficiency reduced AT2 lineage in infected lungs. (**A**) Procedure for the analysis of AT2 lineages using FACS. (**B**) Pseudocolour dot plot for AT2 lineages from H1N1 infection and PBS control mice lungs. (**C**) Scatter dot plot for SPB^+^ AT2 count. 2-tailed Student *t*-test, **** *p* < 0.0001. *n* = 3. (**D**) Scatter dot plot for RAGE^+^ AT1 count. 2-tailed Student *t*-test, * *p* < 0.05. *n* = 3. (**E**) Scatter dot plot for SPB^+^ RAGE^+^ double positive subpopulations count. 2-tailed Student *t*-test, **** *p* < 0.0001; * *p* < 0.05. *n* = 3. Data are presented as mean ± SEM.

To explore how the deficiency of the SPC affects AT2 lineage, both WT and *Sftpc−/−* mice were infected with a sublethal dose of H1N1 virus as previously described [31]. Our FACS results revealed that H1N1 infection stimulated the differentiation of AT2 cells in both groups. This process is essential for replacing damaged AT1 cells and facilitating lung repair. However, a crucial difference emerged between WT and *Sftpc−/−* mice. *Sftpc−/−* significantly reduced AT2 cell differentiation into mature AT1 cells compared to WT controls (Figure 2C,D). Interestingly, we observe an increased AT2 population in *Sftpc−/−* mice, which is due to reduced AT1 differentiation rather than increased AT2 proliferation. Furthermore, H1N1 infection led to a significant increase in double-positive subpopulations of AT2 cells, identified by the presence of both SPB and RAGE markers within *Sftpc−/−* mice lungs (Figure 2E).

### 3.3. Sftpc Gene Regulated AT2 Cell Proliferation and Organoid Colony Formation

**Figure 3.**
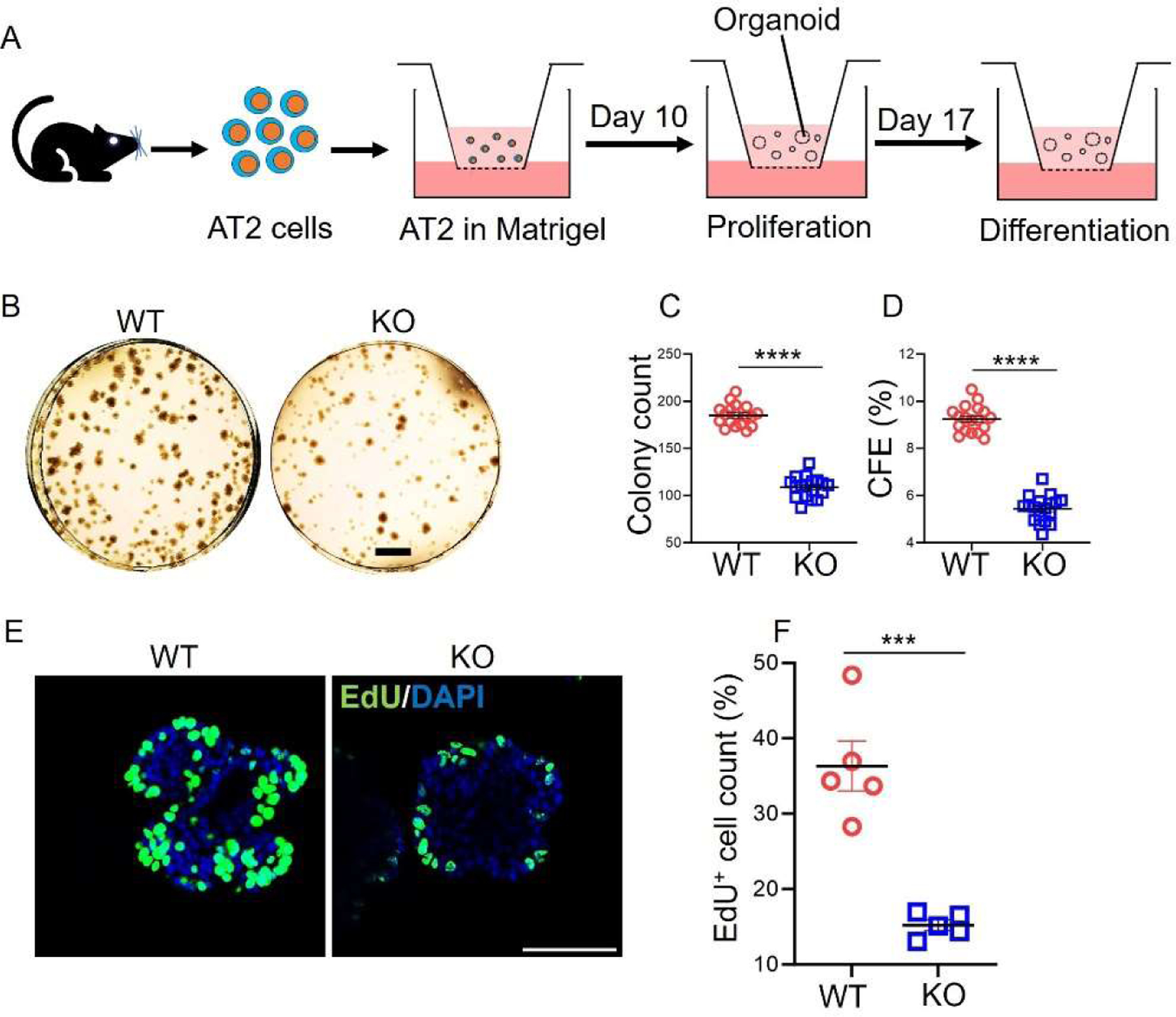
Downregulation of AT2 cell proliferation and organoid colony formation in vitro. (**A**) Procedure of growing feeder-free AT2 organoids in proliferation and differentiation conditions. (**B**) Representative brightfield images of 3D organoids in proliferative mode on day 10. Scale bar: 1 mm. (**C**) Scatter dot plot for colony count on day 10. 2-tailed Student *t*-test, **** *p* < 0.0001. *n* = 18. (**D**) Scatter dot plot for colony forming efficiency on day 10. 2-tailed Student *t*-test, **** *p* < 0.0001. *n* = 18. (**E**) Representative confocal images for EdU incorporation in proliferating AT2 cells. Scale bar: 75 μm. (**F**) Scatter dot plot to compare the number of EdU-positive cells in organoids. 2-tailed Student *t*-test, *** *p* < 0.001. *n* = 5. Images were analyzed using ImageJ software. Data are presented as mean ± SD.

To examine whether the SPC directly influences AT2 cell proliferation, an equal number of AT2 cells from WT and *Sftpc−/−* mice were seeded onto transwell inserts and allowed to form colonies (Figure 3A). We compared the number and efficiency of colony formation between the two groups. Analysis of brightfield images captured on day 10 (Figure 3B–D) revealed a significant reduction in both the total number of colonies (*p* < 0.0001) and colony-forming efficiency (*p* < 0.0001) of AT2 cells from *Sftpc−/−* mice compared to the WT group (Figure 3C,D). We found similar results with our feeder cell co-cultured organoids model. Analysis of differential interference contrast (DIC) images (supplementary Figure S2A) revealed a significant reduction in both the total number of colonies (*p* < 0.0001) and colony-forming efficiency (*p* < 0.0001) of AT2 cells from *Sftpc−/−* mice compared to the WT group images (supplementary Figure S2B,C). These results suggest that *Sftpc−/−* might hinder the ability of AT2 cells to proliferate and form colonies in vitro. To further investigate this effect on AT2 proliferation, an EdU incorporation assay was performed. This assay measures the presence of EdU, a molecule incorporated into DNA during active cell division. The results (Figure 3E,F) confirmed that organoids derived from *Sftpc−/−* AT2 cells contained a significantly lower number of proliferating AT2 cells compared to WT controls (*p* = 0.0003). In conclusion, these in vitro experiments strongly suggest that SPC is critical in AT2 cell proliferation and colony formation.

### 3.4. Sftpc−/− Disrupted AT2 Cell Lineage and Organoid Structure

**Figure 4.**
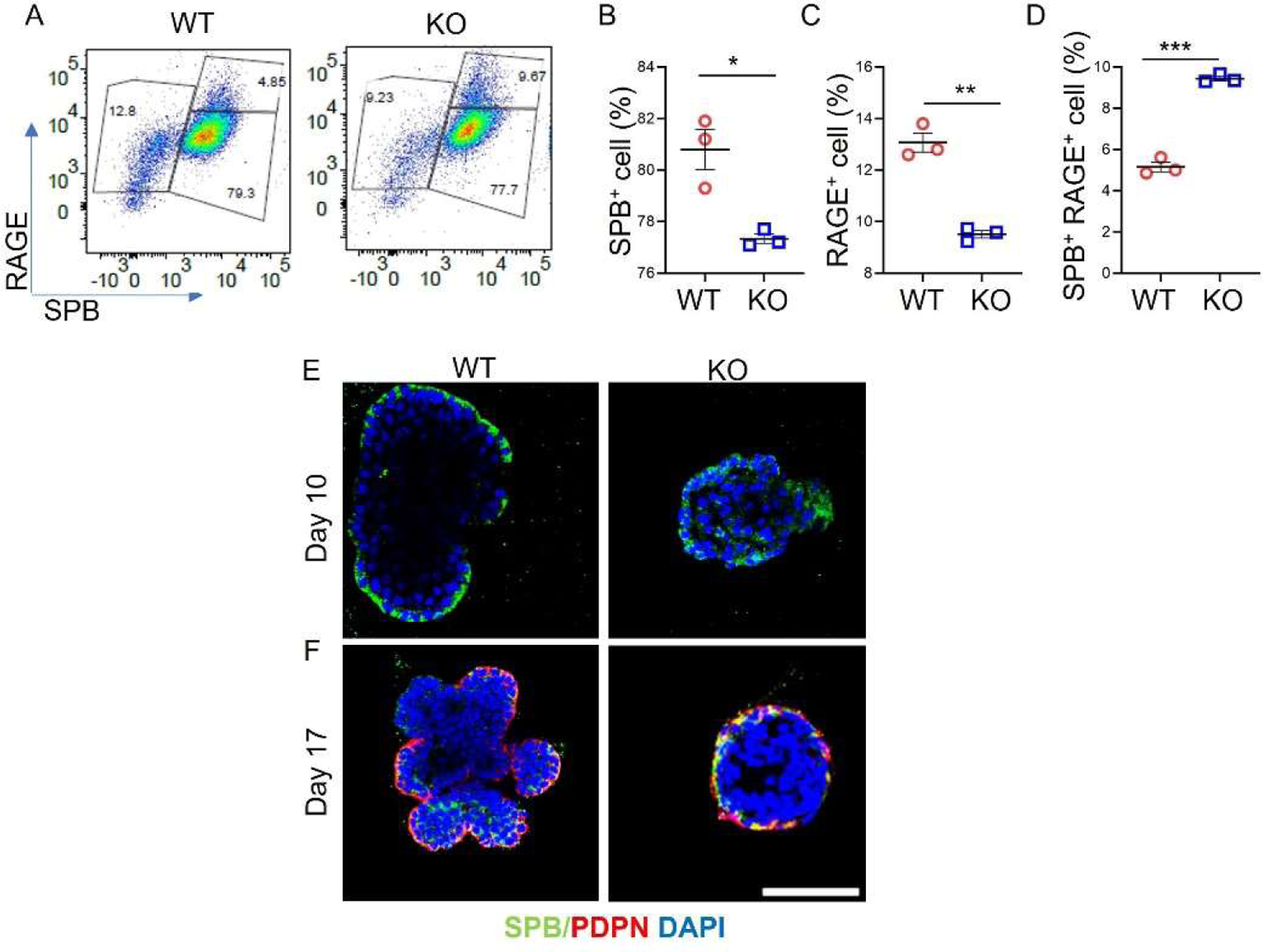
Downregulation of AT2 lineages in *Sftpc−/−* organoids. (**A**) Pseudocolour scatter dot plot for AT2 lineages in WT and *Sftpc−/−* organoids on day 17. (**B**) Scatter dot plot for SPB^+^ AT2 count. 2-tailed Student *t*-test, * *p* < 0.05. *n* = 3. (**C**) Scatter dot plot for RAGE^+^ AT1 count. 2-tailed Student *t*-test, ** *p* < 0.01. *n* = 3. (**D**) Scatter dot plot for SPB^+^ RAGE^+^ transitional cells. 2-tailed Student *t*-test, *** *p* < 0.001. *n* = 3. Data are presented as mean ± SD. (**E**) Representative immunofluorescent confocal images of proliferated organoids on day 10. Scale bar: 75 μm (**F**) Representative immunofluorescent confocal images of differentiated organoids on day 17.

To examine whether the *Sftpc* gene influences AT2 cell lineage, differentiated organoid colonies were dissociated into single cells and labeled with AT2 and AT1-specific antibodies. FACS analysis confirmed that *Sftpc−/−* significantly reduced AT2 (*p* = 0.001) and AT1 (*p* = 0.02) cell counts and increased transitional cells (*p* = 0.0002) count in the *Sftpc−/−* group compared to WT group (Figure 4A–D). Day 10 confocal images revealed large-sized WT organoids with a thin outer layer on the surface, comprised of numerous AT2 cells expressing SPB, a marker for mature AT2 cells (Figure 4E). In contrast, *Sftpc−/−* organoids of small size contained fewer AT2 cells expressing SPB. In differentiated (day 17) WT and *Sftpc−/−* organoids, we observed AT2 cells transitioning into the AT1 lineage in both groups during the differentiation stage (Figure 4F). However, compared to WT controls, more AT2 cells in *Sftpc−/−* organoids appeared as transitional cells. Consequently, fewer AT2 cells successfully differentiated into AT1 cells within *Sftpc−/−* organoids.

### 3.5. Sftpc−/− Upregulated CD74 Expression in AT2 Organoids

**Figure 5.**
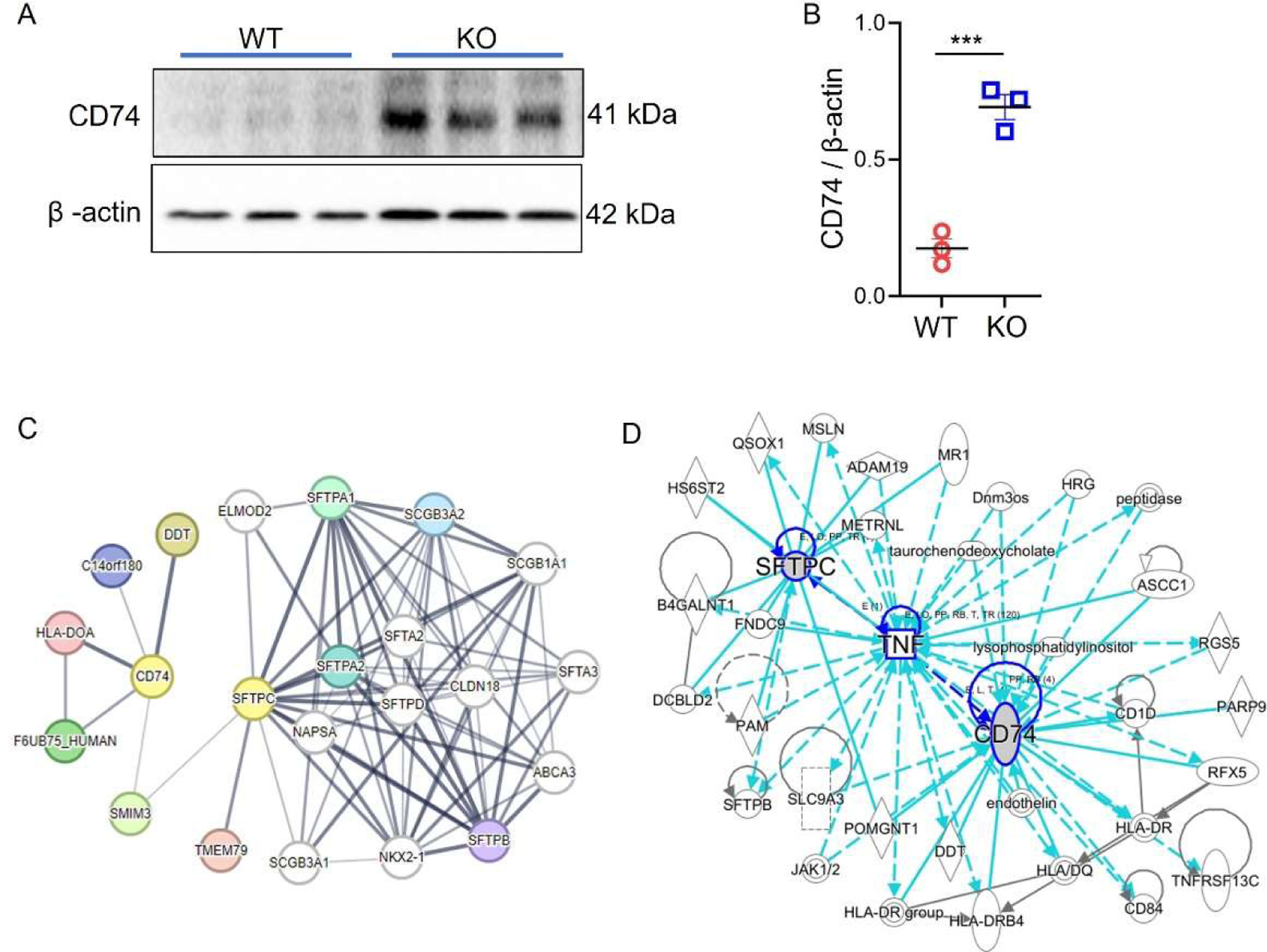
*Sftpc−/−* upregulated CD74 expression in AT2 organoids. (**A**) Representative Western blot of CD74 and loading control β-actin. Blots were cropped to improve the clarity. Full-length blots are presented in Supplementary Figure S6. (**B**) Scatter dot plot for CD74 protein levels in *Sftpc−/−* and WT organoids. 2-tailed Student *t*-test, *** *p* < 0.001. *n* = 3. (**C**) STRING protein network. (**D**) IPA protein network.

We employed Western blot to compare the protein expression level of CD74 between WT and *Sftpc−/−* organoids. Our Western blot analysis revealed a significant upregulation of the protein level of CD74 (*p* = 0.0008) in *Sftpc−/−* organoids compared to WT (Figure 5A,B). We further investigated the potential interaction between CD74 and SFTPC using the STRING database and Ingenuity Pathway Analysis (IPA) analysis (QIAGEN Inc., Hilden, Germany, https://digitalinsights.qiagen.com/IPA) [32]. In the STRING network shown below (Figure 5A), CD74 is connected to several proteins related to immune responses, such as HLA-DOA and DDT. SFTPC, on the other hand, is more involved with surfactant proteins like SFTPA1 and SFTPB. The IPA analysis (Figure 5B) adds further insight by revealing a crucial intermediate link between CD74 and SFTPC via TNF (Tumor Necrosis Factor). TNF acts as a bridge between CD74 and SFTPC, implying that TNF might be a key player in facilitating communication between immune system components (involving CD74) and surfactant proteins (involving SPC).

### 3.6. siRNA Knockdown of CD74 Improved AT2 Colony Formation, AT2 Number and AT1 Differentiation

**Figure 6.**
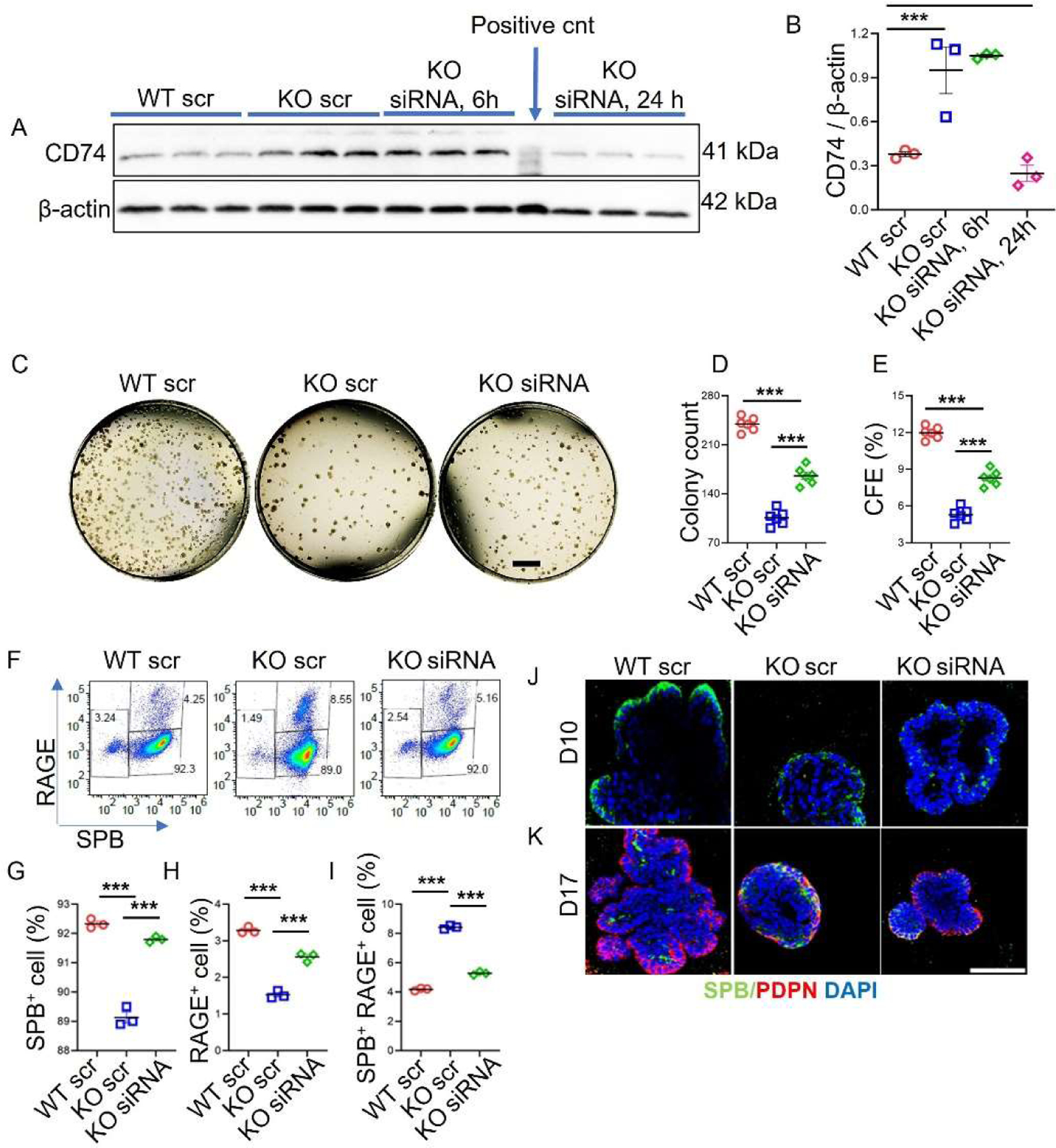
CD74 knockdown increased AT2 organoids forming efficiency, AT2 number, and AT1 differentiation. (**A**) Representative WB of CD74 and loading control β-actin after 24 h siRNA or scramble treatment. BJAB whole cell lysate was used as positive control for CD74 antibody. Full-sized blots are available as Figure S3 in Supplementary Materials. (**B**) Scatter dot plot for CD74 protein. 2-tailed Student *t*-test, *** *p* < 0.001. *n* = 3. (**C**) Representative brightfield images of 3D organoid cultures in proliferative mode on day 10. Scale bar: 1 mm (**D**) Scatter dot plot for colony count on day 10. 2-tailed Student *t*-test, *** *p* < 0.001. *n* = 6. (**E**) Scatter dot plot for colony forming efficiency on day 10. 2-tailed Student *t*-test, *** *p* < 0.001. *n* = 6. (**F**) Pseudocolour scatter dot plot for AT2 lineages in WT scr, *sftpc−*/*−* scr and *sftpc−*/*−* siRNA organoids on day 17. (**G**) Scatter dot plot for SPB+ AT2 cells. 2-tailed Student *t*-test, *** *p* < 0.001. *n* = 3. (**H**) Scatter dot plot for RAGE+ AT1 cells. 2-tailed Student *t*-test, *** *p* < 0.0001. *n* = 3. (**I**) Scatter dot plot for SPB^+^ RAGE^+^ transitional cells AT2 cells. 2-tailed Student *t*-test, *** *p* < 0.001. *n* = 3. Data are presented as mean ± SD. (**J**) Representative immunofluorescent confocal images of proliferated organoids on day 10. Scale bar: 75 μm (**K**) Representative immunofluorescent confocal images of differentiated organoids on day 17.

Organoid-derived AT2 cells were treated with scrambled control or CD74 siRNA for 6 h and 24 h. siRNA treatment for 24 h significantly (*p* = 0.002) reduced CD74 protein expression in *Sftpc−/−* AT2 cells compared to siRNA treatment for 6 h and scramble control treated *Sftpc−/−* AT2 cells (Figure 6A,B). There was no significant difference in the CD74 protein expression between WT scramble and *Sftpc−/−* AT2 cells treated with CD74 siRNA for 24 h. AT2 cells were knocked down for CD74 expression using siRNA for 24 h and then used for colony-forming assay. Equal numbers of scramble control and siRNA-treated cells were seeded in transwells. The colony formation assay revealed that siRNA knockdown of CD74 significantly increases the number of AT2 colonies and colony forming efficiency in *Sftpc−/−* AT2 cells compared to control *Sftpc−/−* AT2 cells (Figure 6C–E). These results suggest the involvement of CD74 in SPC-mediated AT2 cell lineage regulation. FACS results show that CD74 knockdown significantly increased the number of AT2 and AT1 cells and decreased the number of transitional cells in *Sftpc−/−* AT2 organoids compared to control *Sftpc−/−* AT2 organoids (Figure 6F–I). However, this improvement was still significantly lower than WT control, suggesting that SPC protein expression and CD74 knockdown are critical for AT2 lineage function. Confocal immunofluorescence imaging of wholemount organoids confirmed the increased AT2 cell numbers and colony size in CD74 knockdown *Sftpc−/−* proliferating (Day 10) organoids compared to scramble *Sftpc−/−* organoids (Figure 6J). This suggests that CD74 knockdown improves colony-forming efficiency and AT2 cell proliferation in *Sftpc−/−* organoids. In contrast, differentiated organoids showed minimal AT2 cells in WT and CD74 knockdown *Sftpc−/−* organoids compared to scramble *Sftpc−/−* organoids. This indicates that AT2 cells differentiated into mature AT1 cells in both WT and CD74 knockdown *Sftpc−/−* organoids, whereas scramble *Sftpc−/−* organoids contained transitional cells expressing both PDPN and SPB markers (Figure 6K). To validate the effectiveness of siRNA CD74 knockdown in reducing CD74 levels in *Sftpc−/−* organoids and to ensure its sustained efficacy throughout the whole experiment, we performed a Western blot from differentiated organoids (supplementary Figure S4).

## 4. Discussion

This study examined the mechanism of AT2 lineage regulation by the *Sftpc* gene. Our findings demonstrated that the *Sftpc−/−* significantly reduced the total number of AT2 cells compared to WT control lungs. The AT2 cells from *Sftpc−/−* also expressed reduced AT2 and AT1 specific markers, i.e., SPB and PDPN, suggesting a different phenotype of cells. In a previous study, Adam et al. also reported the presence of transdifferentiated cells in pulmonary fibrosis with phenotypes different from AT2 and AT1 cells and possess characteristics of growth arrest, interrupted differentiation, and senescence [33,34]. Our H1N1 infection model demonstrated a difference in response to lung injury between WT and *Sftpc−/−* mice. Here, we demonstrate that the *Sftpc* gene deletion disrupts the AT2 transdifferentiation process, causing AT2 cells to stagnate in the transient states and resulting in reduced counts of both AT2 and AT1 cells. Previous studies have also demonstrated the presence of transitional cells originating from AT2, airway, or club cells in humans and mice after lung injury [34–37]. The transdifferentiation of these transitional cells into AT1 is a critical step for lung repair. The failure of this critical step may potentially impact the lung repair process and induce pulmonary fibrosis. To the best of our knowledge, this is the first study to highlight the critical role of the *Sftpc* gene in regulating the AT2 cell lineage. This impaired response to injury likely makes the lungs of *Sftpc−/−* mice more susceptible to H1N1 infection and impairs the lung repair process. Previous studies have also reported that *Sftpc−/−* mice were more susceptible to infections and bleomycin-induced lung injury compared to WT control mice [8,38–40].

The feeder-free and co-culture (supplementary Figure S2) organoid colony-forming assays displayed a significant reduction in *Sftpc−/−* AT2 colony count and AT2 colony formation efficiency compared to WT control, suggesting reduced proliferating AT2 cells in *Sftpc−/−* mice. Our EdU incorporation assay validated these findings. The small organoid colony size further confirms the reduced proliferative potential of *Sftpc−/−* AT2 cells. According to Choi et al. IL-1β exposure triggers a temporary shift in AT2 cells within organoids towards a progenitor state known as DATPs [37]. This DATP state is critical for the development of mature AT1 cells. However, prolonged exposure to IL-1β in these organoids inhibits AT2 proliferation, leading to an accumulation of DATPs and hindering their differentiation into mature AT1 cells. Our FACS analysis of *Sftpc−/−* organoids exhibited a notably reduced number of AT2 and AT1 cells and a higher number of transitional cells compared to WT controls. This finding suggests that *Sftpc−/−* activates signaling pathways similar to lung injury and disrupts the normal AT2 lineage progression within these organoids, potentially leading to an accumulation of transitional cells AT2 cells.

Previous studies have established CD74 as a marker of AT2 cells, with its expression normally decreasing during AT1 differentiation [41]. Our Western blot analysis of AT2 cells from *Sftpc−/−* mice revealed significantly higher CD74 protein levels compared to wild-type (WT) mice. This study established a link between the *Sftpc* gene and CD74 mediated AT2 lineage regulation. Our findings revealed a significant increase in AT2 proliferation, organoid colony formation, and AT1 differentiation following siRNA-mediated CD74 knockdown in *Sftpc−/−* AT2 cells compared to control *Sftpc−/−* AT2 cells. Based on these observations, we infer that the *Sftpc* gene might regulate AT2 lineage through signaling pathways mediated by CD74. Previous studies have implicated Wnt, Notch, and Hippo pathways in the proliferation and differentiation of AT2 cells [5,42,43]. Further aberrant activity in the Hippo and Wnt pathways has been reported in IPF patients [44]. Our data suggests potential links between the *Sftpc* gene and Wnt5a, Notch3, and Hippo pathways (supplementary Figure S5). While prior studies show CD74 interacts with Notch signaling in the immune system, there is no evidence for this connection in lung repair [45]. Wnt signaling is important for AT2 cells, but there is no direct link yet to how CD74 might influence this pathway in lung repair [28]. Verteporfin disrupts Hippo signaling in AT2 cells, and this study showed that the *Sftpc* gene influences AT2 cells via CD74 receptor [46]. Out of the three pathways, Hippo appears to be the most relevant to CD74.

Current research does not provide a direct link between CD74 knockdown and changes in SPC levels. However, studies on surfactant protein mutations and their effects on AT2 cells suggest that CD74 might play a role in the broader regulatory mechanisms affecting surfactant proteins [47,48]. CD74 knockdown has been shown to influence colony-forming efficiency and cell lineage by affecting proliferation and survival pathways. CD74 interacts with various signaling pathways, including the PI3K/Akt and NF-κB pathways, which are crucial for cell growth and differentiation [49,50]. CD74 knockdown can affect both proliferation and cell death. Studies indicate that silencing CD74 reduces cell proliferation and increases apoptosis, suggesting that CD74 plays a dual role in promoting cell survival and proliferation [51]. CD74 is a receptor for macrophage migration inhibitory factor (MIF). Knockdown of CD74 can disrupt MIF signaling, potentially leading to altered MIF levels and impaired MIF-mediated functions in AT2 cells [52]. Overexpression of CD74 in wild-type AT2 cells could potentially inhibit proliferation and lineage maintenance by altering the balance of signaling pathways that regulate these processes [51,53]. CD74’s interaction with MIF and other signaling molecules might lead to changes in cell behavior, although specific studies on this aspect are limited.

Based on the STRING database, there is no direct interaction between CD74 and SPC, but an indirect connection is present through several intermediate proteins. This suggests that CD74 and SPC are part of different biological processes, but they are interconnected through intermediate proteins involved in both immune response and lung surfactant regulation [54]. However, the direct interaction between CD74 and SPC is not explicitly confirmed by the data provided. The network diagram suggests a relationship, but further experimental validation would be required to confirm a direct protein-protein interaction between CD74 and SPC.

Future studies could investigate how CD74 knockdown affects Notch, Wnt, and Hippo signaling in AT2 cells to shed light on the underlying mechanisms in the context of lung regeneration. Immunofluorescent tracking in vivo is also necessary to assess AT2 to AT1 differentiation directly. Further understanding of these mechanisms could identify potential therapeutic targets for treating ARDS and other lung diseases.

## 5. Conclusions

This study found that CD74 is a valuable regulator of AT2 cell lineage and lung regeneration. We established a link between the *Sftpc* gene and alveolar regeneration, which has not been done before. Our study suggests that the loss of the *Sftpc* gene may drive AT2 cells toward a transitional cells, hindering their ability to proliferate and differentiate effectively. The discovery of CD74 as a regulator for AT2 cell lineage cells opens possibilities for future research into the genetic and cellular mechanisms governing AT2 cell function in development, disease states, and lung repair. Targeting the CD74 receptor could lead to new therapies to promote alveolar repair following the *Sftpc* gene mutations, SARS-CoV-2, and other infection-induced ARDS. Overall, our data suggests potential links between CD74 and Wnt, Notch, and Hippo signaling. However, more research is needed to understand further their interactions with SPC and their specific role in AT2 lineage regulation.

## Supplementary Material

Supplementary Information The following supporting information can be found at https://www.sciepublish.com/article/pii/300, Figure S1. (a) Full size Western blot for SPC expression level in lung tissue, (b) Full size Western blot for SPB expression level in lung tissue, and (c) Full size Western blot for PDPN expression level in lung tissue. Figure S2. Downregulation of AT2 organoid numbers in *Sftpc−/−* mice. A. Representative differential interference contrast (DIC) images of 3D organoid co-cultured with feeder cells. The images were captured on day 10. AT2 cells (5000) were mixed with fibroblast (1 × 10^5^) and cultured in Matrigel to grow 3D organoids in trans-well inserts. B. Scatter dot plot for total organoids colony count. 2-tailed Student *t*-test, **** *p* < 0.0001. *n* = 3. C. Scatter dot plot for colony forming efficiency. 2-tailed Student *t*-test, **** *p* < 0.0001. *n* = 3. Images were analyzed using ImageJ software. Data are presented as mean ± SD. Figure S3. A. Full size Western blot of siRNA mediated CD74 knock down in AT2 cells. B. qRT-PCR of siRNA mediated CD74 knock down in AT2 cells. Figure S4. Full-size Western blot for validation of CD74 siRNA knockdown after 17 days (differentiated) of organoid culture. Figure S5. *Sftpc*−/− may regulate AT2 fate in organoids via Wnt5a, Notch3 and Hippo signaling pathways. Figure S6. Full-size Western blot of CD74 protein expression level in organoid culture. Table S1. Sources and concentration of reagent used in this study.

## Data Availability

All data generated or analyzed during this study are included in this manuscript and its supplementary information files.
