# Supplementary material for: Surfactant Protein-C Regulates Alveolar Type 2 Epithelial Cell Lineages via the CD74 Receptor
Source: J Respir Biol Transl Med. Author manuscript; Available in PMC 2024 Dec 1. (PMC11565471; doi:10.70322/jrbtm.2024.10017)
Supplement: Supplementary Information — The following supporting information can be found at https://www.sciepublish.com/article/pii/300, Figure S1. (a) Full size Western blot for SPC expression level in lung tissue, (b) Full size Western blot for SPB expression level in lung tissue, and (c) Full size Western blot for PDPN expression level in lung tissue. Figure S2. Downregulation of AT2 organoid numbers in Sftpc−/− mice. A. Representative differential interference contrast (DIC) images of 3D organoid co-cultured with feeder cells. The images were captured on day 10. AT2 cells (5000) were mixed with fibroblast (1 × 10^5^) and cultured in Matrigel to grow 3D organoids in trans-well inserts. B. Scatter dot plot for total organoids colony count. 2-tailed Student t-test, **** p < 0.0001. n = 3. C. Scatter dot plot for colony forming efficiency. 2-tailed Student t-test, **** p < 0.0001. n = 3. Images were analyzed using ImageJ software. Data are presented as mean ± SD. Figure S3. A. Full size Western blot of siRNA mediated CD74 knock down in AT2 cells. B. qRT-PCR of siRNA mediated CD74 knock down in AT2 cells. Figure S4. Full-size Western blot for validation of CD74 siRNA knockdown after 17 days (differentiated) of organoid culture. Figure S5. Sftpc−/− may regulate AT2 fate in organoids via Wnt5a, Notch3 and Hippo signaling pathways. Figure S6. Full-size Western blot of CD74 protein expression level in organoid culture. Table S1. Sources and concentration of reagent used in this study. [file NIHMS2029174-supplement-Supplementary_Information.pdf]

Supplementary Materials

Figure S1 (a, b, c). Full size Western blot for (a) SPC, (b) SPB, and (c) PDPN expression level in lung epithelial cells

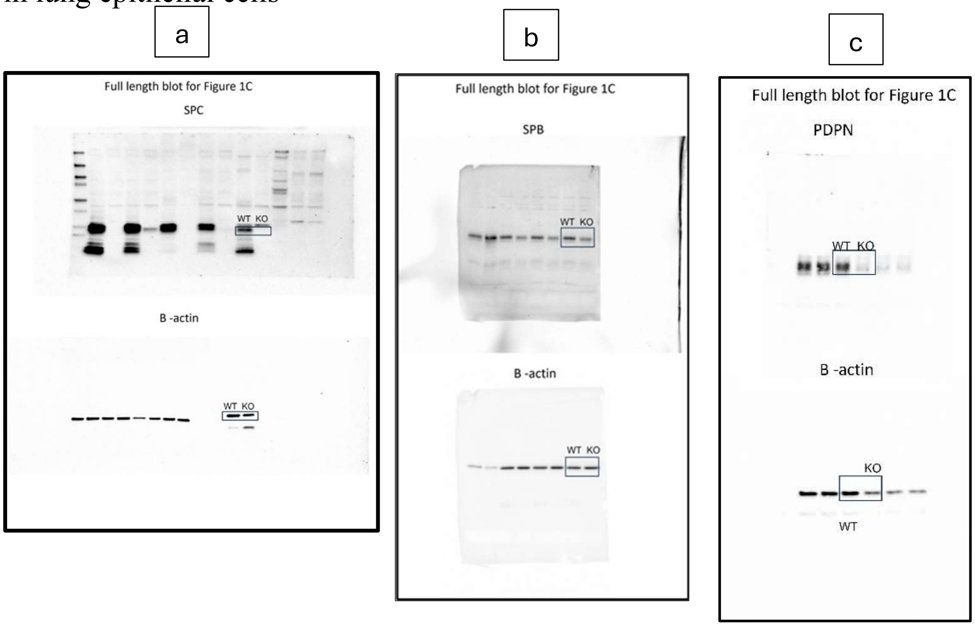

Figure S1 a. Full size Western blot for SPC expression level in lung tissue

Figure S1 b. Full size Western blot for SPB expression level in lung tissue

Figure S1 c. Full size Western blot for PDPN expression level in lung tissue

Figure S2. AT2 organoids co-culture with feeder cells

EPCAM$^+$AT2 cells were isolated from WT and *Sftpc*−/− mice lungs using FACS. The purity and viability of AT2 cells were confirmed, as we described previously (1, 2). AT2 cells (5000) were mixed with mouse lung fibroblasts (MLG2908) (1x10^5) in 60μl Matrigel (1:1 diluted with medium) to culture 3 D organoids in trans well inserts. After 30 min incubation to allow the Matrigel to solidify, 500 μl organoid growth medium was added to the bottom well and further incubated for 4 days. On the fourth day, the medium was switched to the organoid growth medium without an anaplastic lymphoma kinase (ALK) inhibitor, and cultures were maintained for 10 days, with the medium changed every 2 days.

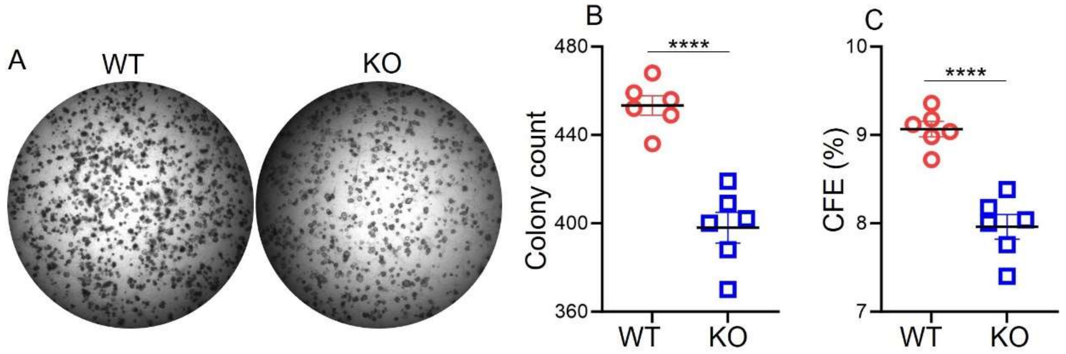

Figure S2. **Downregulation of AT2 organoid numbers in *Sftpc*$^{-/-}$ mice**. A. Representative differential interference contrast (DIC) images of 3D organoid cultures. The images were captured on day 10. AT2 cells (5000) were mixed with fibroblast (1x10^5) and cultured in Matrigel to grow 3D organoids in trans-well inserts. B. Scatter dot plot for total organoids colony count. 2-tailed Student t-test, **** $p < 0.0001$. n=3. C. Scatter dot plot for colony forming efficiency. 2-tailed Student t-test, **** $p < 0.0001$. n=3. Images were analyzed using ImageJ software. Data are presented as mean ± SD.

Figure S3. siRNA mediated CD74 knock down in AT2 cells.

A. Western blot

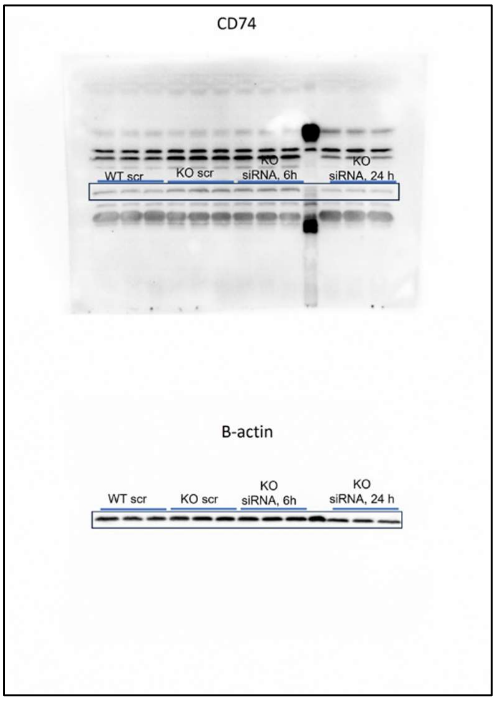

B. qRT-PCR

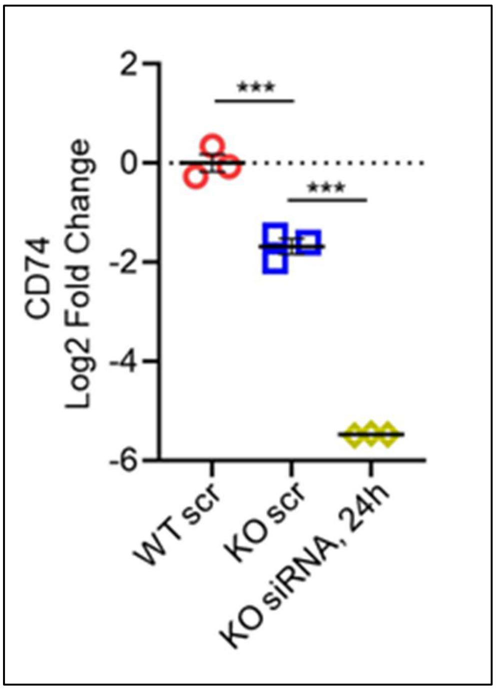

Figure S3. A. Full size Western blot for CD74 siRNA knock down in AT2 cells B. The log2 fold change in CD74 mRNA expression following siRNA knockdown. Please note that the correlation between expression levels of protein and mRNA in mammals is relatively low, with a Pearson correlation coefficient of approximately 0.40 (3, 4). Our RT-qPCR analysis of AT2 cells from *Sftpc*−/− mice revealed significantly lower CD74 mRNA levels compared to wild-type (WT) mice. However, protein analysis showed a surprising reversal, with significantly higher CD74 protein expression in AT2 cells from *Sftpc*−/− mice compared to WT. There may be various reasons for this discrepancy between mRNA and protein expression levels, including post-transcriptional processes and protein stability, as reported previously in the literature. Studies have shown that protein levels are more conserved than

mRNA levels, and that changes in transcription are associated with translational changes that exert opposite effects on the final protein level (5). This is why we reported changes in CD74 protein rather than mRNA expression. Several studies have reported this discrepancy between mRNA and protein levels in cells (6).

Figure S4. Full size Validation of CD74 siRNA knock down after 17 days (differentiated) of organoid culture.

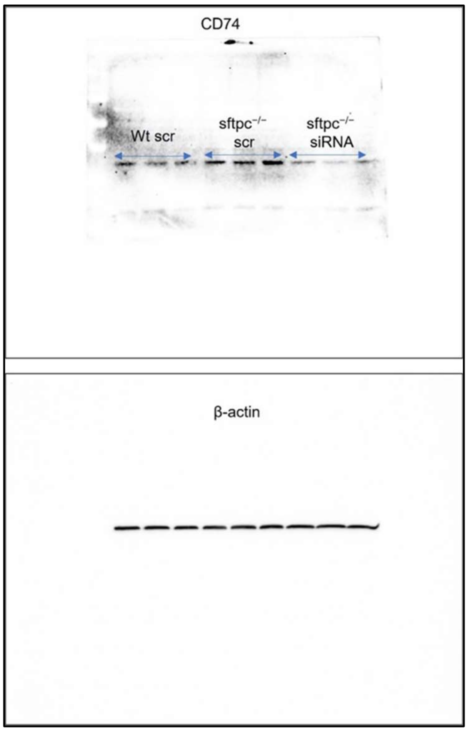

Feeder-free organoids were cultured from siRNA or scramble-treated AT2 for 10 days. siRNA and scramble were added in culture medium on day 10 to continue inhibition of CD74 expression for further 7 days. Western blot image showed that CD74 expression was reduced in siRNA-treated *Sftpc*−/− organoids compared to scramble-treated *Sftpc*−/− organoids.

Figure S5. ***Sftpc***$^{-/-}$ **may regulate AT2 fate in organoids via Wnt5a, Notch3 and Hippo signaling pathway**

Feeder-free *Sftpc*−/− AT2 organoids were treated with an activator or inhibitor of Wnt, Notch and Hippo signaling pathways. Stock solutions of Wnt5a and Notch3 were prepared in PBS and of Box5, verteporfin (VTPF) and DAPT were prepared in DMSO. AT2 organoids were treated with Wnt5a (100ng/mL), Notch3 (100ng/mL), DAPT (50uM), Box5 (100uM) and verteporfin (VTPF) (4uM) and with PBS or DMSO vehicle controls from day 0 to day 10. Treatment of organoids with Notch3 significantly increased colony counts, and treatment with DAPT significantly reduced colony counts. This suggests that *Sftpc*−/− inhibits Notch3 signaling in AT2 cells. Incubation of organoids with Wnt5a increased the number of proliferating colonies but did not affect the count

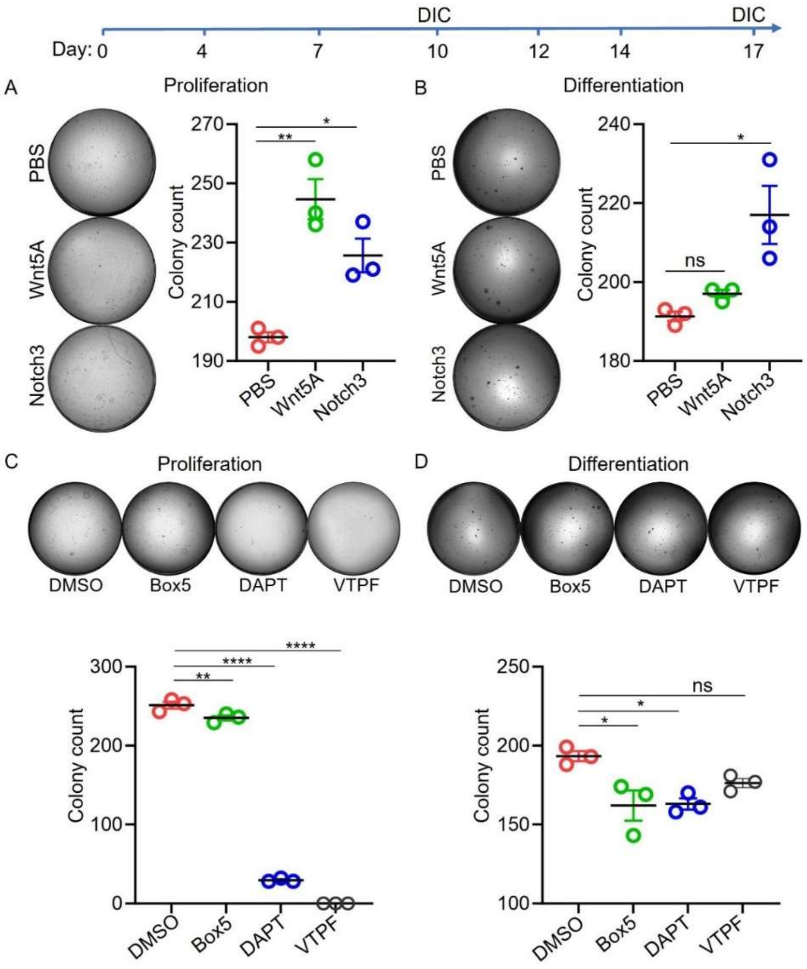

of differentiated colonies. This suggests that *Sftpc*−/− does not affect the Wnt5a pathway in differentiating AT2 cells. Incubation of organoids with verteporfin (an inhibitor of YAP-TEAD complex) significantly reduced both proliferating and differentiating AT2 colonies, suggesting regulation of the YAP pathway by the *Sftpc* gene.

Figure S5. ***Sftpc*$^{-/-}$ may regulate AT2 fate in organoids via Wnt5a, Notch3 and Hippo signaling pathway**. A. Representative differential interference contrast (DIC) images of organoids and scatter dot plot for total organoid colonies number after PBS, Wnt5A (p=0.0013) and Notch3 treatment (p=0.0167) on day 10 in proliferative mode. Unpaired two-tailed ordinary one-way ANOVA, Dunnett test, ** $p < 0.01$, * $p < 0.05$. n=3. B. Representative DIC images of organoids and scatter dot plot for total organoid colonies number after PBS, Wnt5A (p=0.5848) and Notch3 treatment (p=0.0105) on day 17 in differentiative mode. Unpaired two-tailed ordinary one-way ANOVA, Dunnett test, ^{ns} $p > 0.05$, * $p < 0.05$. n=3. C. Representative DIC images of organoids and scatter dot plot for organoid colonies number after DMSO, Box5 (p=0.0086), DAPT (p<0.0001) and VTPF (p<0.0001) treatment on day 10 in proliferative mode. Unpaired two-tailed ordinary one-way ANOVA, Dunnett test, **** $p < 0.0001$, ** $p < 0.01$. n=3. D. Representative DIC images of organoids and scatter dot plots for organoid colonies number after DMSO, Box5 (p=0.0103), DAPT (p=0.0123) and VTPF (p=0.1451) treatment on day 17 in differentiation mode. Unpaired two-tailed ordinary one-way ANOVA, Dunnett test, **** $p < 0.0001$, ** $p < 0.01$. n=3. Images were analyzed using ImageJ software. Data are presented as mean ± SD. Working concentrations, Wnt5a 100ng/mL, Notch3 100ng/mL, DAPT 50uM, Box5 100uM, verteporfin (VTPF) 4uM.

Figure S6. Full size CD74 protein expression level in organoid culture

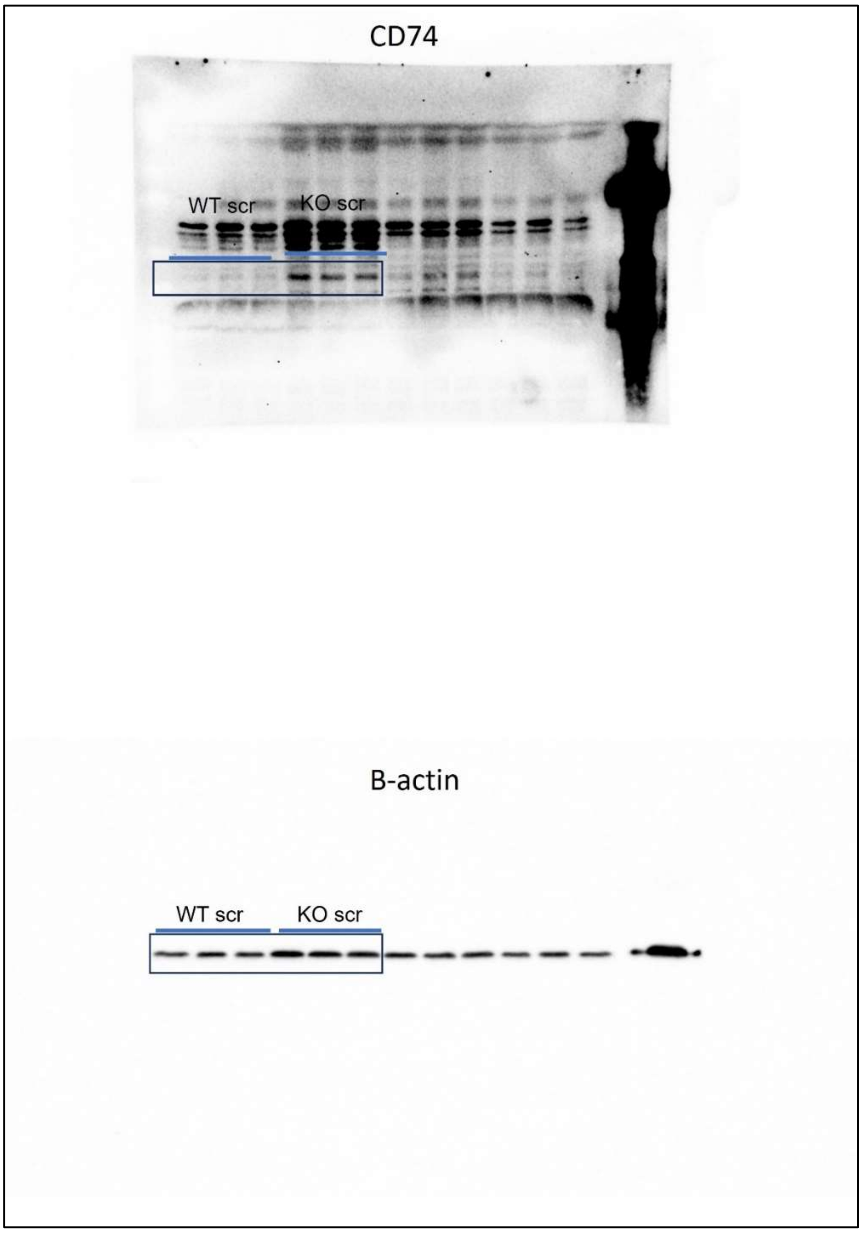

Table S1. Sources and concentration of reagent used in this study.

Kit / Reagent / antibody name	Working concentration	Vendor & Cat#
Biotin conjugated CD16/32	0.65 μg/10^6 cells	BD Biosciences #553143
Biotin conjugated CD45	1.5 μg/10^6 cells	BD Biosciences #553078
Biotin conjugated TER119	1 μl/10^6 cells	BD Biosciences #553672
Dynabeads	2.5 μg/10^6 cells	Invitrogen; #65601
True stain Fc blocker	1 μg/10^6 cells	BioLegend™ #101320
Anti CD326/EPCAM ab, AF488	1 μg/10^6 cells	BioLegend # 118210
7AAD solution	5 μl/ 100 μl	BD Biosciences # 559925
Growth factor reduced Matrigel		Corning, #354230
Anti-SPB Rabbit polyclonal antibody	1:1000	ThermoFisher # PA5-42000
Anti-PDPN Syrian hamster antibody	1:1000	Invitrogen MA516113
Anti-proSPC rabbit antibody	1:500	Millipore #AB3786,
Anti CD74 mouse antibody	1:1000	Santa Cruz biotech #SC-6262
Anti-β-actin monoclonal antibody	1:1000	Santa Cruz #sc-47778
HRP conjugated goat anti-mouse IgG	1:10000	Jackson ImmunoResearch #115-035-147
HRP conjugated anti-rabbit IgG	1:10000	Jackson ImmunoResearch #211-032-171
HRP conjugated goat Anti-Syrian Hamster IgG	1:10000	Jackson ImmunoResearch # 107-035-142
AF 488 goat anti-rabbit IgG (H+L)	1:500	Jackson Immuno #111-545-045
AF647 Goat anti-Syrian Hamster IgG (H+L)	1:500	Invitrogen #A-21451,
Click-iT 5-ethynyl-2-deoxyuridine (EdU) assay kit	As per kit instruction	Thermofisher # C10499
RNA easy micro kit	As per kit instruction	Qiagen #74004

iScript™ Reverse Transcription Supermix	As per kit instruction	Bio-Rad #1708840
SYBR green	10µL/20µL reaction	Bio-Rad #1725270
CD74 siRNA	80pmol	Santa Cruz biotech #SC-35024
Scramble control siRNA	80pmol	Santa Cruz biotech #SC-37007

References

1. Ali G, Zhang M, Zhao R, Jain KG, Chang J, Komatsu S, et al. Fibrinolytic niche is required for alveolar type 2 cell-mediated alveologenesis via a uPA-A6-CD44(+)-ENaC signal cascade. Signal Transduct Target Ther. 2021;6(1):97.
2. Zhao R, Ali G, Chang J, Komatsu S, Tsukasaki Y, Nie HG, et al. Proliferative regulation of alveolar epithelial type 2 progenitor cells by human Scnn1d gene. Theranostics. 2019;9(26):8155-70.
3. Vogel C, Marcotte EM. Insights into the regulation of protein abundance from proteomic and transcriptomic analyses. Nature Reviews Genetics. 2012;13(4):227-32.
4. Kosti I, Jain N, Aran D, Butte AJ, Sirota M. Cross-tissue Analysis of Gene and Protein Expression in Normal and Cancer Tissues. Scientific Reports. 2016;6(1):24799.
5. Perl K, Ushakov K, Pozniak Y, Yizhar-Barnea O, Bhonker Y, Shivatzki S, et al. Reduced changes in protein compared to mRNA levels across non-proliferating tissues. BMC Genomics. 2017;18(1):305.
6. Greenbaum D, Colangelo C, Williams K, Gerstein M. Comparing protein abundance and mRNA expression levels on a genomic scale. Genome Biol. 2003;4(9):117.
